# First records and description of metallic red females of *Euglossa* (*Alloglossura*) *gorgonensis* Cheesman, with notes on color variation within the species (Hymenoptera, Apidae)

**DOI:** 10.3897/zookeys.335.6134

**Published:** 2013-09-25

**Authors:** Ismael A. Hinojosa-Díaz, Berry J. Brosi

**Affiliations:** 1 Department of Environmental Studies, Emory University, Math and Science Center, 5th Floor, 400 Dowman Drive, Atlanta, Georgia 30322, USA

**Keywords:** Apoidea, *Euglossa*, orchid bees, Costa Rica, color variation

## Abstract

Metallic coloration is one of the signatures of orchid bees of the genus *Euglossa*, with some species showing variation associated with their geographic range. *Euglossa (Alloglossura) gorgonensis* Cheesman exhibits color variation, ranging from mainly green specimens in the southern extreme of its range (Pacific slope of Colombia), to noticeably reddish specimens in parts of the northern known limits of its range (Pacific slope of southern Costa Rica). Here we present the first description of females from Costa Rica belonging to the reddish extreme of the color variation.

## Introduction

Besides their interesting biology, orchid bees are morphologically attractive, among other things due to the metallic coloration of the body. Within the genus *Euglossa* Latreille in particular, with few exceptions (see Hinojosa-Díaz and Engel 2011a), species exhibit bright metallic coloration all over the body, including colors such as green, blue, bronze–reddish (see Roubik and Hanson 2004), variations of these, as well as combinations and intergradations of them. A number of species in the genus are known to be variable in the metallic coloration of the body, which can be associated with the geographic range of the particular species. Examples are discussed by Roubik (2004) for species in the subgenus *Glossura* Cockerell, and by Hinojosa-Díaz and Engel (2012) for species in the recently proposed subgenus *Alloglossura*. As part of this last assemblage, *Euglossa (Alloglossura) gorgonensis* Cheesman, exhibits integumental color variation along its distributional range, from predominantly green specimens in the southern Pacific slope of Colombia, to distinctively reddish specimens in the southern Pacific slope of Costa Rica (Hinojosa-Díaz and Engel 2012). The species was originally described based on green females from Gorgona Island in Colombia, while Dressler (1978) created a separate subspecies for the reddish males from Costa Rica. Until now females for the red extreme of the color variation were unknown. Here we present a description of females from the Pacific slope of southern Costa Rica filling this knowledge gap.

## Material and methods

The female specimens used in this study were collected as part of a study of the effects of forest fragmentation on Costa Rican bee communities (Brosi et al. 2007, Brosi et al. 2008, Brosi 2009). One specimen was captured via aerial netting in a bee survey; the other was captured in a Van Someren trap baited with rotten fish. One of the specimens is housed in B. Brosi’s collection at Emory University, Atlanta, Georgia, USA, the other is deposited in the Division of Entomology, University of Kansas Natural History Museum, Lawrence, Kansas, USA (SEMC).

Morphological terminology in general follows that of Engel (2001), and Michener (2007); some procedures for establishing metrics (e.g., clypeal protuberance) follow Brooks (1988). Length measurements are presented as the average of the two specimens used in the study with individual specimen measurements in parenthesis, except when both specimens had the same value. The description is based on the overall format for *Euglossa* species as presented by Hinojosa-Díaz and Engel (2007, 2011a, 2011b, 2012). Photomicrographs were prepared using a Cannon EOS 7D digital camera and an Infinity K-2 long–distance microscope lens. Multilayer images were produced by using the software CombineZP.

## Results

### Euglossa (Alloglossura) gorgonensis

Cheesman

http://species-id.net/wiki/Euglossa_gorgonensis

Figs 1–3

Euglossa gorgonensis Cheesman, 1929: 141–154 [146].
Euglossa (Glossura) gorgonensis erythrophana Dressler, 1978: 167–185 [170].

#### Female red morph description.

**Figures 1–3.**
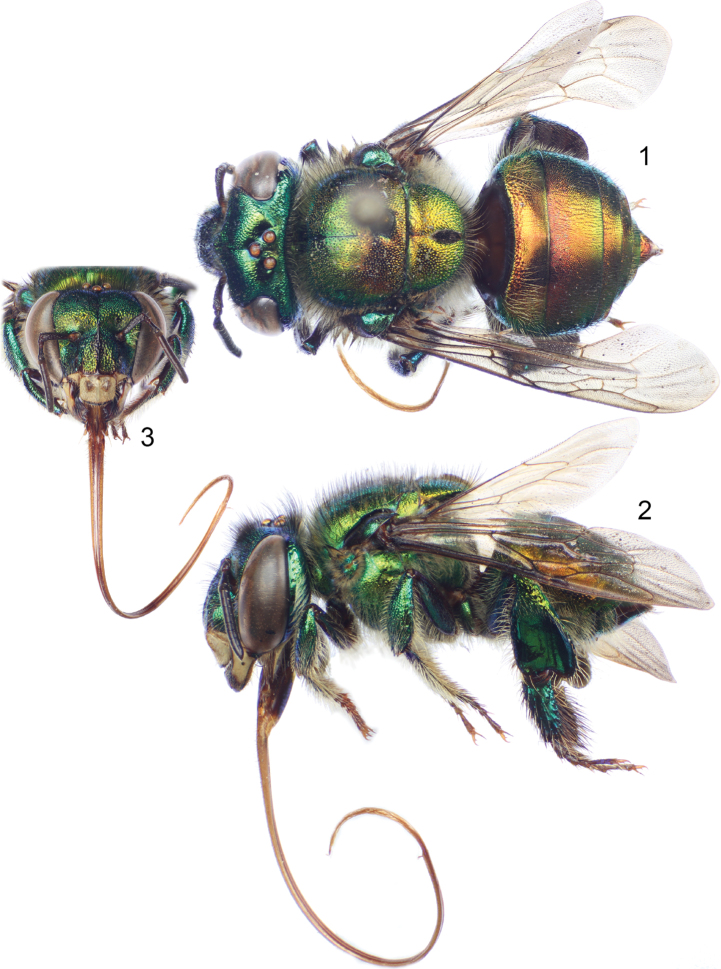
*Euglossa (Alloglossura) gorgonensis* Cheesman, female, red specimen from the Pacific slope of southern Costa Rica. **1** Dorsal habitus **2** Lateral habitus **3** Facial aspect.

*Structure*. Total body length 10.45 mm (10.30, 10.59); labiomaxillary complex in repose slightly surpassing metasomal tip (estimation) (Figs 1-2). Head length 2.63 mm (2.56, 2.70); head width 4.23 mm (4.16, 4.30); upper interorbital distance 2.15 mm; lower interorbital distance 2.04 mm (2.01, 2.07); upper clypeal width 1.23 mm (1.19, 1.26); lower clypeal width 1.84 mm (1.83, 1.85); clypeal protuberance 0.78 mm (0.74, 0.81); medial clypeal ridge well developed, paramedial clypeal ridges well developed along their lower two thirds; labrum slightly wider than long, length 0.98 mm (0.96, 1.00), width 1.11 mm (1.10, 1.11); medial labral ridge sharp; paramedial labral ridges sharp, oblique, running on about four fifths of labral length; labral windows occupying about half of labral length; interocellar distance 0.33 mm; ocellocular distance 0.66 mm; length of first flagellomere [0.39 mm (0.37, 0.41)] comparable to combined length of second and third flagellomeres [0.39 mm (0.37, 0.41)]; length of malar area 0.05 mm. Mandible tridentate. Pronotal lateral angle characteristic of *Alloglossura* (slightly obtuse not broadened anterolaterally and with no projections); intertegular distance 3.26 mm; mesoscutal length 2.69 mm (2.59, 2.78); mesoscutellar length 1.30 mm (1.26, 1.33); posterior margin of mesoscutellum strongly convex; mesotibial length 2.04 mm (2.00, 2.07); mesobasitarsal length 1.86 mm (1.78, 1.93), maximum width 0.50 mm (0.48, 0.52); metatibia triangular (scalene triangular), metatibial anterior margin sinuate, proximally concave, length 2.78 mm (2.67, 2.89); ventral margin length 1.78 mm (1.56, 2.00); metatibial posterodorsal margin length 3.12 mm (3.04, 3.19); metabasitarsus characteristic of *Alloglossura* females (trapezoidal with narrower and straight distal margin) (Fig. 2), length 1.56 mm (1.48, 1.63), maximum width 0.72 mm (0.70, 0.74). Forewing length 8.04 mm (7.78, 8.30); hind wing with 17–20 hamuli. Maximum metasomal width 4.26 mm (4.22, 4.30).

*Coloration*. Head green, with noticeable golden–bronzy iridescence all over but accentuated on frontal areas (clypeal disc, and antennal depressions); some blue lights on vertex and lower paraocular areas; clypeal disc with brown coloration as in previously known specimens (i.e. restricted to contiguous areas along upper half of medial ridge) (Fig. 3); mesosoma green, with golden–bronzy iridescence all over, turning reddish on posterior half of mesoscutum, as well as on all mesoscutellar surface; legs green, with golden–bronzy iridescence; metasoma with basal green coloration overtaken by strong golden–reddish iridescence, particularly dominant on dorsal surfaces of terga (Figs 1–2).

*Sculpturing and vestiture*. As described for the known predominantly green specimens (i.e. Hinojosa-Díaz and Engel 2012).

#### Material examined.

Both female specimens from Costa Rica, labeled as follows: “Specimen’04 # 2251; morphospp: 94 // Tucanes [vertical writing] Costa Rica, Coto Brus, near San Vito; 8°49'01.88"N, 82°59'31.02"W BG; elev. 1200m; Aug. 2004; aerial netting; B.Brosi, T.Shih, B. Graham IXT 8/25” (1♀) (Emory University); “Practice #94; morphospp: // Santa Clara [near San Vito de Coto Brus, Puntarenas, Costa Rica, 8°48'23.56"N, 82°58'35.02"W] 7/9; FISH Int // c.f. Euglossa; gorgonensis // Collected outside of sampling; regime; morphospp #: 94 [captured in the middle of a 3–Ha forest fragment using a Van Someren trap baited with rotten fish, on 9-July-2004]“(1♀) (SEMC).

## Discussion

Cheesman (1929) originally described *Euglossa (Alloglossura) gorgonensis* based on two female specimens from Gorgona Island, off the southern Pacific coast of Colombia. The holotype specimen (and presumably the other female of the type series) exhibits a characteristic green integument with some blue–green iridescence, and very faint golden–bronzy lights. A couple of additional females, from the Canal Zone in Panama, included in the redescription of the species as part of the subgenus *Alloglossura* (Hinojosa-Díaz and Engel 2012), are similarly colored with the exception of the golden–bronzy iridescence being more noticeable, especially on the anterior section of the metasomal terga (see Hinojosa-Díaz and Engel 2012, figs 36–37). On the other hand, male specimens of *Euglossa (Alloglossura) gorgonensis*, are known from Colombia, Panama and Costa Rica. As asserted by Hinojosa-Díaz and Engel (2012), specimens from Colombia and Panama have corresponding integumental coloration to the females as mentioned above (i.e. mainly green with weak golden–bronzy iridescence). The Costa Rican male specimens exhibit a range of integumental coloration with dominant golden–bronzy iridescence that turns intense red in specimens from some areas of the southern Pacific slope of the country. The distinctive bright red iridescent coloration of these males seemed to be compelling enough to describe a subspecies, *Euglossa (Alloglossura) gorgonensis erythrophana* Dressler (1978), distinguishable from the predominantly green specimens. Following Hinojosa-Díaz and Engel (2012), the subspecific names within *Euglossa (Alloglossura) gorgonensis* are seen as synonymous, as there seems to be continuous intergradation from the bright red specimens in the southern Pacific side of Costa Rica, to the rather green specimens from the southern Pacific of Colombia. Despite this intergradation, the red colored specimens are quite distinctive and females had been unknown until now. The two females used for the present work were collected in localities in which bright red males have also been collected; these localities are also in the proximity of localities cited in the original description of *Euglossa (Alloglossura) gorgonensis erythrophana*. The discovery of these female specimens is significant from more than one point of view. They fill up a gap in terms of the knowledge of the morphological variation of the species. The females here studies are distinctively red colored, more noticeably on the posterior dorsal half of the mesosoma, and more strongly on all metasomal terga. The red coloration is not as strong as in some of the males from the region (see Hinojosa-Díaz and Engel 2012, figs 34–35), but they are distinctive from the Panamanian and Colombian previously known female specimens. There is also variation in the coloration of the males in the area, all of them having the distinctive bright red integument, but to different degrees, some of them matching the coloration of the females here studied. It should be noted, as stated by Hinojosa-Díaz and Engel (2012), that *Alloglossura* femalestend to exhibit more extended blue–green to purple coloration than the males. Another interesting aspect of the discovery of these females lies in the general scarcity or absence of female specimens in collections from the area. Despite the constant surveying of orchid bees in both Costa Rica and Panama, females are unknown for other species, like the closely related *Euglossa (Alloglossura) oleolucens* Dressler (see Hinojosa-Díaz and Engel 2012). The bias towards the collection of males by using chemical baits could explain the paucity of female specimens, which are rarely attracted to these baits; however both authors have spent more than one season collecting bees from flowers in the region, having collected no females of *Euglossa (Alloglossura) oleolucens*, and in the case of a longer survey by B.J. Brosi, the two females of *Euglossa (Alloglossura) gorgonensis* here presented. One of these two females was captured in a trap baited with rotten fish, which could be an indication of the biology of the species. We encourage the use of alternative collecting methods in addition to the use of chemical baits, to sample the orchid bee fauna as a way to get a better picture of the morphological variation with respect to the female bees.

## Supplementary Material

XML Treatment for Euglossa (Alloglossura) gorgonensis
